# Behavioural Repertoire of Working Donkeys and Consistency of Behaviour over Time, as a Preliminary Step towards Identifying Pain-Related Behaviours

**DOI:** 10.1371/journal.pone.0101877

**Published:** 2014-07-30

**Authors:** Fran H. Regan, Jo Hockenhull, Joy C. Pritchard, Avril E. Waterman-Pearson, Helen R. Whay

**Affiliations:** 1 School of Veterinary Sciences, University of Bristol, Langford, North Somerset, United Kingdom; 2 Animals in International Development, Banwell, North Somerset, United Kingdom; 3 The Brooke, London, United Kingdom; University of Rennes 1, France

## Abstract

**Background:**

The donkey has a reputation for stoicism and its behavioural repertoire in clinical contexts is under-reported. Lack of understanding of the norms of donkey behaviour and how it may vary over time can compromise use of behavioural measures as indicators of pain or emotional state. The objective of this study was to find out whether the behaviour of working donkeys was influenced by gender, the time of day or differed between days with a view to assessing how robust these measures are for inclusion in a working donkey ethogram.

**Methodology/Principal Findings:**

Frequency and consistency of postural and event behaviours were measured in 21 adult working donkeys (12 females; 9 males). Instantaneous (scan) and focal sampling were used to measure maintenance, lying, ingestive and investigative behaviours at hourly intervals for ten sessions on each of two consecutive days. High head carriage and biting were seen more frequently in male donkeys than females (P<0.001). Level head carriage, licking/chewing and head-shaking were observed more frequently in female donkeys (P<0.001). Tail position, ear orientation, foot stamping, rolling/lying and head-shaking behaviours were affected by time of day (P<0.001). However, only two variations in ear orientation were found to be significantly different over the two days of observations (P<0.001). Tail swishing, head shaking, foot stamping, and ears held sideways and downwards were significantly correlated (P<0.001) and are assumed to be behaviours to discourage flies.

**Conclusions/Significance:**

All donkeys expressed an extensive behavioural repertoire, although some differences in behaviour were evident between genders. While most behaviours were consistent over time, some behaviours were influenced by time of day. Few behaviours differed between the two test days. The findings can be used to inform the development of a robust, evidence-based ethogram for working donkeys.

## Introduction

Donkeys working in developing countries are used for transporting both goods and people, often in extreme heat and humidity, in urban working environments where there are hazards such as heavy traffic, noise, pollution and debris. Working donkeys are usually owned by very poor members of society and seldom receive adequate resources and management to maintain good welfare. During rest periods, donkeys often remain tethered or harnessed alone or in pairs. Consequently, they have little opportunity to engage in truly restorative resting behaviour or to socialise freely.

Domestic donkeys are descended from the African wild ass [1]. Observations of wild ass and populations of feral donkeys provide an insight into donkey behaviour outside of the working environment. The donkey digestive system is very similar to that of the horse, and if unimpeded donkeys will spend a comparable amount of their time grazing, typically 14–16 hours a day [2]. The social organisation of wild ass and feral donkeys can vary with their environment and the resources available [1], [3], [4], with some populations operating resource or territory guarding systems and others guarding females or harems [3]. Although the social lives of working donkeys have yet to be studied, the propensity for donkeys to form pair-bonds or situation –specific groupings demonstrates their social tendencies [5] and lack of opportunity to engage in social behaviour may therefore compromise their welfare further.

Behavioural observation gives the most reliable and immediate insight into the animal's perception of and interaction with its environment [6]. Behavioural divergence from the norm may be clinically significant, for example may indicate pain or pathology. It is likely that many working donkeys suffer from pain associated with multiple acute and/or chronic clinical conditions. Donkeys behave differently to horses [7], and while a number of equid ethograms exist, e.g. [8], they typically focus exclusively on horse behaviour. Little is published regarding the behavioural repertoire of the domestic donkey, particularly under working conditions. As a result, it is challenging to identify when donkey behaviour changes from the norm, compromising the use of behaviour as a welfare indicator in this species.

Research investigating consistency of behavioural parameters has examined natural behaviour and treatment effects in a range of species including horses [9], dairy cows [10], red deer [11] and lambs [12]. Consistency of behaviour can be defined as a percentage agreement between behavioural observations over time. Studying the influence of time on behaviour enables researchers to identify the behaviours most suitable for measurement during short observation periods, making them practical for use in clinical situations.

Diurnal variations in the behaviour of different groups of horses and ponies have previously been investigated, for example see [13] and [14], studies of Przewalski's horse and New Forest ponies respectively. Few peer reviewed studies could be found on diurnal patterns or consistency of measured behaviours in domestic donkeys, although detailed studies of diurnal variations in activity [15] grazing behaviour [16] and physiological measures [17] in small samples of donkeys have been published. Posture and event behaviours have been used in livestock research to assess pain [12], [18], fear [19], [20], distress [21], anxiety and adaptation to novel environments or stimuli [22]. The lack of information regarding the normal behaviour of donkeys hinders progress towards recognition of abnormal behaviour indicative of pain, fear or distress. Improving our understanding of what constitutes normal postural and event behaviours for donkeys will enable us to detect more readily when behaviour diverges from the norm, and facilitate the use of behaviour as an indicator of pain and emotional state in this species.

The study objective was to find out whether the posture and event behaviours recorded in working donkeys were influenced by gender or time of day and whether they differed between consecutive days. In addition, an avoidance test was used to gauge reaction towards approaching humans [23].

## Materials and Methods

### Ethics statement

The study was carried out in Lahore, Pakistan during November 2005, under ethical approval from the University of Bristol Faculty of Medical and Veterinary Sciences Ethics Committee (Investigation number UB/05/017) and was compliant with Pakistan law regarding ethical use of animals in science.

### Donkey selection and acclimatisation to study environment

Twenty-one working donkeys were recruited from within a 15 km catchment area of Shadara fodder market, Lahore. Inclusion criteria for the study included: non-pregnant females, entire males, minimum body condition score of 1.5 (1 (very thin) - 5 (very fat) [24], donkeys over 3 yrs of age but under 10 years, no acute traumatic injury or severe chronic injury, no wounds or minor body wounds only, no obvious lameness, and intact ears and nostrils. Bodyweight was recorded using an electronic weighbridge (Ezi-weigh, Tru-test Ltd, New Zealand). Donkeys were loose-housed individually in 3 m×3 m shaded pen enclosures with both physical and visual contact with another donkey. They were bedded on dry straw, offered water *ad libitum* and a food ration twice daily of crushed grains and chopped roughage formulated to aid malnourished animals. An acclimatisation period of 18 h enabled animals to recover from any acute stress, fear, hunger or thirst resulting from their daily working lives and familiarised them with the environment prior to starting behavioural observations.

### Behavioural observation period

The ethogram used in this study (see Table 1) was developed through pilot studies with working and non-working donkeys and included all possible behaviour that a donkey might display [25]. Behavioural observations were made for ten minutes at hourly intervals (excluding 12:00) for ten sessions on two consecutive days. Behaviours were recorded directly onto a check sheet by a single observer (FHR) seated 3.5 metres outside the pen. Observations consisted of:

**Table 1:** Ethogram of behaviours observed in domestic (including working) donkeys at rest* organised by the observation type used in the current study.

Category	Behaviour	Description
*Pen Position*	Back of pen	Head and forelimbs in back half of pen (irrespective of orientation)
	Front of pen	Head and forelimbs in front half of pen (irrespective of orientation)
**Postural Behaviours (instantaneous (scan) sampling):**		
***Lying down***	Lying sternally	Lying down on sternum, legs folded underneath body frame
	Lying laterally	Lying on side with legs outstretched, head and neck may be in contact with ground
***Standing***	Standing on 4 feet	Weight-bearing on all 4 limbs with no preferred loading
	Standing on 3 feet	Weight-bearing on 3 limbs with a hind limb resting
	Pointing	Placing a foot in a forwards position outside of the main body frame (minimum 1 hoof length) with reduced weight-bearing
	Knuckled	Forwards bend of one or both fore limbs with the knee bent in front of the placed foot or fetlock joint
***Ear position***	Forwards	Both ears facing forwards with ear cups fully visible when facing the donkey head-on
	Sideways	Both ears facing sideways with ear cup orientated approximately 90 degrees laterally from forwards-facing position
	Backwards	Both ears facing backwards with ear cups visible when standing behind the donkey
	Combinations	Each ear in a different orientation, e.g. one facing forwards, one facing backwards
***Ear level***	Down	Tip of ear level with or below level of base of ear, drooping downwards in any orientation
	Up	Tip of ear above base of ear, in any orientation
***Head carriage***	High	Poll higher than top of withers
	Level	Poll level with top of withers
	Low	Base of ears below top of withers
	Very low	Nose contact with ground
***Head direction***	No turn	Straight head carriage, no turning of head towards self, object or stimuli
	Turn to belly	Head is turned towards or makes contact with either side of abdomen
	Turn to flank	Head is turned towards or makes contact with either flank
	Turn to limb/foot	Head is turned towards or makes contact with either fore/hind limb/foot
	Turn to look	Head is orientated away from a straight position with attention drawn towards environmental stimuli and not directed towards a body part/region
***Tail position***	Relaxed	Tail held in a relaxed position, hanging freely in a vertical line from its body base
	Lifted out	Tail held in a fixed position, sticking out more than 45 degrees from the vertical line
	Tucked	Tail held tightly against the rump in a fixed position, with tip of tail tucked between hind legs
	Swishing	Tail moves swiftly from its base in a side-to-side flicking manner around the hind quarters
**Event behaviours (focal sampling):**		
***Body***	Rolling	From lying down laterally or sternally, vigorous rolling and wriggling movement of whole body over onto back
	Transition up/down	From lying down laterally or sternally to standing, or vice versa
***Feet***	Walking	Forwards, backwards or sideways movement of limbs to a new position
	Pawing	Repetitive lifting and backwards dragging or scraping of pointed hoof
	Leg lifting	Temporary lifting of any limb from the ground with hesitant replacement near to its original position, often repetitive
	Weight shifting	Weight temporarily off-loaded from a fore or hind limb onto the remaining 3 limbs, accompanied by subtle body rock
	Foot stamping	Sudden lifting and forceful replacement of any limb in its original position
***Oral***	Eating	Prehension of food into the mouth, with repetitive chewing and swallowing
	Drinking	Muzzle touches water source followed by at least 1 visible swallow
	Sniffing	Movement of muzzle towards object, ground or body, followed by inhalation and movement of nostrils
	Flehmen response	Upper lip curls back to expose gums with incisors meeting, head tips back and rapidly points muzzle upwards
	Licking and chewing	Repetitive licking and chewing motion in the absence of any food in the mouth
	Yawning	Mouth opens wide, eyes close, head rises, tips back and shakes, lower jaw grinds and closes
	Biting**	Grasps object, self or another donkey in open mouth and bites or chews with single or repeated jaw closures
***Head***	Head shaking	Vigorous rotational shake of head and neck resulting in ears flapping against sides
	Head tossing	Rapid up and down movements of neck with successive nods of head
***Vocal***	Snorting	Quick forced exhalation of air through nostrils making an audible noise
	Braying	Series of short duration, loud inhalations, followed by a prolonged noisy exhalation
	Sighing	Prolonged deep inhalation with a noticeable rise of the body, followed by a short burst of expiration, then gradual release of air
	Groaning	Similar to a sigh, but accompanied by a monotone prolonged vocalization and often head is turned towards body part
	Coughing	Distinctive short duration exhalation from the lungs, deep and often moist in sound, often accompanied by a body heave
***Maintenance***	Self grooming	Repeated grooming movement of mouth and incisors directed at own body parts
	Rubbing	Moving a body part against another (e.g. eye rubbing on fore leg) or repeatedly moving a body part back and forth against any object
	Stretching	Head tucks into neck, mouth opens slightly, eyes often close, whole body contracts and rises up noticeably with inhalation
	Dozing	State of body stillness with eyes closed, head lowered and standing on all 4 feet, or standing on 3 feet and resting a leg
***Eliminative***	Defaecating	Elimination of faeces
	Urinating	Elimination of urine
***Play***	Playing	Lifting up and tipping/ holding objects with mouth, rearing, broncing, bucking or attempted interaction

*NB: the ethogram contains behaviours that may be observed in domestic donkeys, and is not limited to those seen in working donkeys and/or those that were possible in the context of this study.

**The donkeys in this study did not have physical access to other donkeys during the observation period; therefore biting in this context refers to biting objects or self.

- instantaneous (scan) sampling at one minute intervals throughout each ten minute observation period, recording the anatomical components of posture (ear orientation, head carriage, feet position, and tail carriage), general activity category (standing, walking, lying, rolling, and eating) and the donkey's location in the pen (front or back; standing at the front of the pen would allow visual contact with other donkeys) at the scan time point.
- focal sampling of pre-defined event behaviours, such as snorting, braying and leg lifting, recording all occurrences throughout the 10 minute period. Some event behaviours were recorded if a single occurrence was observed (for example cough, snort, leg-lift), and others if the behaviour continued for a minimum of 3 s (such as licking/chewing, body-rubbing, walking).

The ethogram (Table 1) is organised to reflect the two types of observations made in the study.

### Avoidance tests

An avoidance test was developed by adapting measures of fear in livestock [26], [27]. Following pilot testing, score system [27] was modified so that the observer (FHR) approached each donkey, loose within its pen, from a distance of 3 meters by walking slowly towards the side (shoulder area) of the donkey avoiding direct eye contact. The stage at which the donkey withdrew was then scored (for description of scores see Table 2). The avoidance test was carried out once during the acclimatisation period and twice a day at 12 hour intervals (before and after behavioural observations) on the two consecutive test days.

**Table 2:** Descriptors used to score reaction to the avoidance test in working donkeys, modified from [22].

Score	Description
−1	Donkey does not allow observer to enter pen, aggression displayed
0	Donkey withdraws at observer's approach (1 metre)
1	Donkey allows observer to approach but withdraws at shoulder touch
2	Donkey allows observer to touch shoulder but withdraws at head touch
3	Donkey allows observer to touch head and does not withdraw

### Statistical Analysis

All data were converted into total number of observations of each posture or event behaviour per 10 minute observation period and per day for each donkey. Data were not normally distributed, so non-parametric tests were used for all analyses. Data were tested for significant gender differences using the Mann-Whitney U test. Friedman analysis of variance (ANOVA) with chi-squared statistic was used to investigate the effect of time of day on observed behaviours. The Wilcoxon Signed Rank test was used to test for differences between matched observation periods on day 1 and day 2. Kendalls coefficient of concordance was used to look for associations within groups of behaviours hypothesised to be related to each other. Statistical analysis was performed using SPSS version 19.0 (SPSS Inc., Illinois, USA) and the significance level was set at *P*<0.05. Holm's sequentially rejective multiple test procedure [28] was used to reduce the risk of Type I errors through the multiple tests conducted, with only the variables meeting the adjusted P value denoted by the procedure retaining their significance and being reported as such.

## Results

### Body condition and body weight

The mean +/− s.d. age of the 21 donkeys in years was 5.7+/−1.7. Mean bodyweight was 120 kg+/−19 and the median body condition score was 2: thin (min = 1 very thin, max = 2.5 thin/medium). There were no significant differences in bodyweight (Mann Whitney U = 43.5; median male 116.5 kg; median female 118 kg; P = 0.464), age (Mann Whitney U = 53; median male 5 years; median female 6 years; P = 0.972) and body condition score (Mann Whitney U = 56.5; median male 2; median female 1.75; P = 0.862) between male and female donkeys.

### Behaviour

During the observation period the donkeys stood for a median (min-max) of 78.4% (48.2–94.5%) of the sampling points. Walking was observed for a median of 5.7% (1.6–13.4%) and lying for 12.3% (0–40%) with some donkeys not seen lying at all. A median of 56.6 (20.0–84.0) occurrences of eating behaviour were observed throughout the period. Maintenance behaviours, such as rolling, self-grooming and stretching were rarely observed with medians of 0.9 (0–6.8%), 3.5 (2–10 occurrences) and 0 (0–1.5 occurrences) respectively.

### Behaviour: effect of gender

Table 3 shows behaviours which differed significantly between male and female donkeys. Standing, walking, lying, rolling, head turning, tail position and location in pen were not influenced by gender. Some patterns were found with head carriage: female donkeys held their heads in the level position (poll level with withers) (Mann-Whitney U = 339.5; P<0.001) more frequently than male donkeys. Male donkeys more frequently held their heads in the high position (poll above withers) (Mann-Whitney U = 39.0; P<0.001), indicating a more vigilant posture. Lying and maintenance behaviours were unaffected by gender. Biting (Mann-Whitney U = 120.0; P<0.001) was more frequently demonstrated by male donkeys while head-shaking (Mann-Whitney U = 325.0; P<0.001) and licking/chewing were seen more frequently in females (Mann-Whitney U = 387.0; P<0.001).

**Table 3:** Postural and event behaviours significantly more frequently observed in male (n = 9) or female (n = 12) working donkeys (bold cells denote gender where the behaviour was most frequently observed).

Behaviour	Mann-Whitney U statistic *(significance, p)*	Male donkeys *Median (min-max)*	Female donkeys *Median (min-max)*
**Postural behaviours:**			
High head carriage	39.0 (<0.001)	**18.18 (0–100)**	4.54 (0–90.91)
Level head carriage	399.5 (<0.001)	18.18 (0–90.91)	**40.91 (0–100)**
**Event behaviours:**			
Licking and chewing	387.0 (<0.001)	0 (0–5)	**1 (0–10)**
Head shaking	325.0 (<0.001)	0 (0–9)	**1 (0–12)**
Biting object/self	120.0 (<0.001)	**0 (0–13)**	0 (0–0)

### Behaviour: effect of time

Table 4 shows the behaviours which were significantly affected by the time of day. Tail position (relaxed, tucked and swishing) and ear orientation (one ear forwards, one sideways; one sideways, one backwards; and both ears sideways and facing down) differed significantly across the day (Friedman χ^2^ =  from 33.8 to 204.3; P<0.001). Lying down/rolling and foot stamping were also significantly affected by time of day. The majority of event behaviours were consistent over time, with the exception of head-shaking which was influenced by time of day (Friedman χ^2^ = 91.7; P<0.001).

**Table 4:** Postural and event behaviours which differed in frequency within a single day in 21 working donkeys.

Behaviour	Number of observations for all time points on 2 consecutive days *Median (min-max)*	Difference in frequency of observations between 10 time points across a single day *Friedman ANOVA with Chi squared statistic (significance, p)*
**Postural behaviours:**		
***General***		
**Lying down/rolling**	0 (0–95.45)	36.869 (<0.001)
***Tail position***		
**Relaxed**	47.73 (0–100)	111.502 (<0.001)
**Tucked**	0 (0–100)	41.011 (<0.001)
**Swishing**	40.91 (0–95.45)	204.296 (<0.001)
***Feet position***		
**Foot stamping**	0 (0–22.73)	39.346 (<0.001)
***Ear orientation***		
**Combination: FF/SS**	0 (0–22.73)	40.431 (<0.001)
**Combination: SS/BB**	0 (0–22.73)	33.831 (<0.001)
**Combination: SD/SD**	0 (0–100)	51.276 (<0.001)
**Event behaviours:**		
**Head shaking**	0 (0–12)	91.692 (<0.001)

Ear orientation: FF/SS  =  one ear forwards, one sideways; SS/BB  =  one ear sideways, one backwards; SD/SD  =  both ears sideways and facing down.

Only two measures were found to significantly differ across the two test days: both ears sideways (Wilcoxon −3.945; median Day 1 = 18.18, median Day 2 = 13.64; P<0.001) and both ears sideways and down (Wilcoxon 3.602; median Day 1 = 18.18, median Day 2 = 22.73; P<0.001). All other postural behaviours and event behaviours were consistent across the test days.

Using the Kendall's coefficient test, foot-stamping, tail swishing, ears held sideways facing down and headshaking were all positively associated with each other (R = 0.780, P<0.001).

### Avoidance scores

Three female donkeys did not let the observer enter the pen to start the test on each test session and so scored −1 for each test. For those that did let the observer start the test, avoidance scores did not differ significantly with time of day (Wilcoxon 1242.0; median am = 3, median pm = 3; P = 0.242) or the day of testing (Wilcoxon 1714.0; median day 1 = 3, median day 2 = 3; P = 0.184). There was a significant difference in avoidance test scores between genders (Mann-Whitney U = 1162.5; median male = 3, median female = 2; P = 0.003)

## Discussion

The results of this study confirm that donkeys have an extensive behavioural repertoire, as found in previous studies [25], although some behaviours are observed much more frequently than others. Several behaviours varied between male and female donkeys and this should be accounted for when assessing behavioural signs of pain in working donkeys. Some behaviours of working donkeys are not consistent over time, which may make them unsuitable for use in a tool for recognising pain behaviour in clinical situations.

The donkeys spent the majority of the observational period standing with very little time spent on maintenance behaviours. Gender-specific patterns for a number of postural and event behaviours were found, with the strongest differences seen in head carriage. In male donkeys high head carriage and biting were seen, suggesting a focus on the external environment. These are components of a normal social and sexual behaviour repertoire [29], [30] and are indicative of the male donkeys' strong motivation to investigate surroundings and interact with con-specifics. Female donkeys appeared to be less focused on the external environment than the males, expressed by their level head carriage. Headshaking was significantly more frequent in females and may suggest that they react more vigorously than male donkeys to the presence of flies. No comparable published behavioural research could be found, therefore the results of this study can only be considered in the context of this study group and environment.

This study has also helped refine the working donkey ethogram by investigating changes in behaviours over time, enabling inclusion of the behaviours which are most robust and useable in the field at any time of day. The majority of foot and limb positions, head carriage positions and event behaviours were found to be consistent over time. Strong time-dependent effects on headshaking, foot stamping and tail swishing were hypothesised to be due to the presence of flies, which was greatest in the middle of the day, although in this study fly levels were not recorded directly. This supports the finding that free ranging Przewalski's horses increase their activity and feeding budgets at night compared to daytime levels in the summer months, due to high temperatures and disturbance by flying insects [13]. Lying down/rolling varied significantly across the eleven hour observation period. In a study of adult feral donkeys in California lying posture during daylight hours in hot months was never observed, suggesting that donkeys may change their lying behaviour to reduce the risk of heat stress [30]. The donkeys in the present study also spent a relatively short time lying down during the daylight observation periods; although they were observed informally to be recumbent during hours of darkness. Working donkeys are rarely allowed to rest un-harnessed and have little or no opportunity to lie down during a typical day, which may contribute to chronic fatigue.

Only two variants of ear orientation were found to differ significantly across test days, with the remaining postural and all event behaviours proving to be consistent. This provides strong evidence for the robustness of these behaviours over time and their inclusion in a working donkey ethogram.

Behavioural observations in the current study were limited to daylight hours; pilot studies using video-recording of behaviour at night were unsuccessful due to an erratic power supply. Observation of behaviour over only two consecutive days was a further limitation caused by the need to return donkeys to their owners after a relatively short period of time. However, the behaviour observed would not be expected to change markedly given an extended observation period.

The avoidance scoring method generated results comparable with previous research. The present study found that 57% of donkeys achieved the top score (allowing their head to be touched by an approaching observer), in agreement with the 56% of working donkeys rated in a previous study [24] as either non-responsive or eliciting a friendly response. The present study found that 24% of donkeys showed a negative reaction (aggression towards or avoidance of the test observer), fewer than the 44% of donkeys reported by [24] although still a cause for concern. This could be due to the difference in test environments: donkeys in the current study were loose in a pen, having rested, eaten food and received gentle handling (i.e. avoiding practices commonly seen during the donkey's working day such as shouting, slapping and hitting the donkeys with sticks) prior to testing, while the donkeys in the previous study described above [24] were scored as found during their working day. A consistent negative avoidance test score (−1) was exhibited by three of the 21 donkeys in the present study and the observer could not safely enter the pen to start the test. Prolonged or persistent fear can be a serious compromise to an animal's welfare and can result in depression, chronic fatigue, restlessness and anorexia [31]. Female donkeys scored significantly lower in the test than males and the three donkeys that consistently had a negative score in the avoidance tests were all female. Gender differences in emotional reactivity have been identified in horses, with mares being more easily panicked and aggressive than geldings [32]; our findings suggest similar gender differences in donkeys.

This study has confirmed that working donkeys have a large behavioural repertoire and identified some behaviours which lack diurnal consistency in their expression. Few behaviours varied significantly across the test days indicating that time of day has a greater impact on behaviour observed than the day itself. This should be taken into consideration in future observational studies.
